# When good citizens do bad things for their organization: examining the link between organizational citizenship behavior and unethical pro-organizational behavior

**DOI:** 10.3389/fpsyg.2026.1711730

**Published:** 2026-05-08

**Authors:** Fubin Xia, Ping Lu, Lifang Wang

**Affiliations:** 1 School of Economics and Management, Hanshan Normal University, Chaozhou, China; 2 School of Education Science, Hanshan Normal University, Chaozhou, China; 3 School of Business Administration, Dongbei University of Finance and Economy, Dalian, China

**Keywords:** ego depletion, job insecurity, OCB, self-regulatory, UPB

## Abstract

**Introduction:**

Organizational citizenship behavior (OCB) is generally regarded as a positive form of extra-role behavior that supports organizational effectiveness. However, engaging in OCB also requires self-regulatory effort, which may make employees more prone to unethical pro-organizational behavior. Drawing on the resource model of self-control, this study examines when and why OCB may have this unintended effect.

**Methods:**

A moderated mediation model was employed to investigate the impact of OCB on unethical pro-organizational behavior. The hypothesized framework was tested using two-wave survey data drawn from 376 employees in Chinese enterprises.

**Results:**

The results show that OCB has a significant positive effect on unethical pro-organizational behavior. Ego depletion partly explains this relationship, indicating that engaging in OCB consumes employees' self-control resources, which in turn increases the likelihood of unethical pro-organizational behavior. Moreover, this indirect relationship becomes stronger when job insecurity is high.

**Discussion:**

These findings reveal the paradoxical nature of OCB. Although it is generally regarded as beneficial, it may also entail ethical costs. The study therefore offers a more nuanced understanding of discretionary behavior and its potential ethical risks.

## 1 Introduction

The relationship between employees and the organization is a symbiotic one. Due to this symbiotic relationship, employees often make extra contributions to the organization beyond their job duties, such as working overtime and voluntarily helping their colleagues. These extra-role, discretionary behaviors are commonly referred to as organizational citizenship behavior (OCB). Smith et al. (1983) introduced this concept, and Organ (1988) further characterized it as discretionary employee behavior that goes beyond formal role requirements, is not explicitly recognized by the reward system, and nonetheless supports the effective functioning of the organization. Employees who exhibit OCB are regarded as good citizens (Bergeron, 2007). Beyond its definitional emphasis as a discretionary extra-role behavior, OCB has been widely recognized as beneficial to organizational effectiveness and as a potential source of strategic advantage for organizations (Bolino and Grant, 2016). Employees are more likely to engage in OCB when they work in organizations that have social responsibility (e.g., Ong et al., 2018; Tourigny et al., 2019; Gullifor et al., 2023), fair leadership (e.g., Mo and Shi, 2017; Gerpott et al., 2019; Jun et al., 2024), supportive co-workers (e.g., Balliet and Ferris, 2013; Feys et al., 2013; Patterer et al., 2024). However, the role-exceeding behavior of good citizens is not without cost, and requires extra physiological and psychological effort. Existing studies have found that after engaging in OCB, employees suffer from physical and mental impairments (Bolino et al., 2015), reduced task performance (Bergeron et al., 2013, 2014), and other problems due to the additional effort required. This implies that good citizens who care about and support the organization are willing to bear these costs, voluntarily sacrificing themselves for the sake of the organization.

Good citizens may not always sacrifice themselves for the organization. Recent studies have revealed that good citizens who exhibit OCB may also engage in inappropriate work behavior, including counterproductive behavior (Dalal et al., 2009; Klotz and Bolino, 2013; Sulistiawan et al., 2025), deviant behavior (Yam et al., 2017), knowledge hiding (He et al., 2024), and unethical behavior (Bolino and Klotz, 2015) that harm the organization's interests for their own personal gain. However, some good citizens may also resort to negative work behaviors not for personal gain, but to help the organization (Umphress et al., 2010). Specifically, some good citizens may, for certain reasons, feel compelled to overextend themselves and engage in unethical acts for the benefit of the organization. For example, an employee may exaggerate product benefits, withhold unfavorable information from customers, or distort facts to protect the organization's interests. These behaviors reflect unethical pro-organizational behavior (UPB), which refers to unethical actions undertaken to benefit the organization or its members (Umphress et al., 2010; Umphress and Bingham, 2011). Therefore, this study aims to explore the mechanisms through which OCB influences UPB, uncovering the underlying reasons and conditions for the behavioral change of good citizens.

We analyze this underexplored dark aspect of OCB based on the resource model of self-control (Muraven and Baumeister, 2000; Baumeister et al., 2024). OCB is a behavior that consumes personal resources (Bolino and Grant, 2016), and according to the resource model of self-control (Muraven and Baumeister, 2000; Baumeister et al., 2024), good citizens may experience ego depletion due to the consumption of personal resources while engaging in OCB. Research has shown that the depletion of self-control resources weakens individuals' moral awareness, making them more likely to resort to unethical means to achieve their goals (Mead et al., 2009; Gino et al., 2011). Accordingly, when citizens lack sufficient personal resources to sustain their engagement in organizational citizenship behaviors, they may—in order to continue supporting the organization—resort to unethical behaviors that require minimal effort and self-regulatory exertion.

Furthermore, this situation may be influenced by employees' perceived job insecurity. Job insecurity is the subjective perception of employees regarding the stability of their current job and their interpretation of the current work environment as a sense of threat (Sverke et al., 2002; Shoss, 2017). Employees who consistently fulfill their job responsibilities and proactively engage in extra-role behaviors often view the organization as a critical platform for self-actualization. Consequently, they devote substantial cognitive and temporal resources to sustaining its operations over extended periods. This deep investment renders them particularly susceptible to workplace threat cues, which they are more prone to interpret as a fundamental challenge to their professional worth and past contributions, thus amplifying perceived job insecurity. In such contexts, they may intensify their OCB in an effort to gain recognition and acceptance from the organization. This, in turn, further depletes their self-regulatory resources. As these cognitive and emotional resources become increasingly exhausted, the prolonged experience of job insecurity may weaken individuals' moral restraints, thereby heightening the likelihood of engaging in UPB as a compensatory strategy to signal continued loyalty and commitment to the organization.

In summary, this study examines how and when good citizens engage in unethical behaviors to benefit the organization, using the self-control resource model. It offers a novel lens on OCB's adverse consequences and provides insights for mitigating its unintended organizational impacts.

## 2 Theory and hypothesis development

### 2.1 OCB and UPB

UPB is intentional conduct that employees engage in to promote the effective functioning of the organization or its members, while violating the core values of society, moral customs, laws, and norms of proper conduct (Umphress et al., 2010; Umphress and Bingham, 2011). We argue that good citizens may engage in such behavior not due to malicious intent, but as a reluctant choice driven by self-control failure, in order to continue helping the organization. Self-control is a basic human ability that enables individuals to perform socially expected behaviors; however, exercising self-control consumes personal resources (Inzlicht and Schmeichel, 2012). The resource model of self-control highlights that human cognitive resources are finite and readily depleted. As individuals expend cognitive resources in the process of self-regulation, these resources may become insufficient or depleted, leading to self-control failure (Muraven and Baumeister, 2000). Individuals experiencing self-control failure struggle to prevent themselves from engaging in socially unacceptable behaviors, such as unethical conduct (Gino et al., 2011; Weiss et al., 2021). Good citizens need to exert willpower to perform organizational citizenship behavior, which is expected by the organization and consumes considerable personal resources (Bolino and Grant, 2016). Based on the resource model of self-control, when good citizens experience resource depletion as a result of OCB, they may find it difficult to maintain their previous level of moral self-regulation. They may also lack the necessary self-control resources to continue supporting the organization as they once did. Under such conditions, in an effort to sustain their contributions, they may overlook ethical standards and adopt less cognitively demanding strategies to achieve this goal. Compared with OCB, which require sustained personal investment and conscientious effort, UPB are a cognitively economical but morally compromised means of supporting the organization. Therefore, good citizens may become more inclined to engage in UPB as a consequence of the diminished self-regulatory capacity resulting from their involvement in OCB. Based on this rationale, we propose hypothesis 1.

*H1:* OCB is positively related to UPB.

### 2.2 Mediating effect of ego depletion

Ego depletion is a temporary state of resource deficiency that occurs after the consumption of personal resources (Hagger et al., 2010). Good citizens need to consume more personal resources to perform OCB (Nielsen et al., 2012). For example, helping colleagues requires good citizens to understand their needs, provide a help suggestion or plan, communicate it to them, and answer any questions or confusion they may have. Similarly, providing suggestions to the organization requires good citizens to research the problems existing in the organization, collect, record and analyze information, attend meetings and make reports, and seek help from the organization. Therefore, as good citizens continue to perform OCB, they consume more and more personal resources. Although individuals can recover from resource depletion through various ways such as rest or sleep (Lanaj et al., 2014; Litwiller et al., 2017; Liu et al., 2021), if recovery is delayed or consumption consistently exceeds replenishment, good citizens may experience ego depletion due to sustained deficits. This risk may be particularly pronounced in organizational environments that encourage continuous citizenship behaviors without offering corresponding support or recovery mechanisms. It can thus be concluded that OCB and ego depletion are positively correlated. On the basis of this possibility, Hypothesis 2 is proposed.

*H2:* OCB is positively associated with ego depletion.

In the workplace, research has shown that ego depletion can lead to employees engaging in negative work behaviors. Ego depletion impairs self-control abilities and reduces the willingness to exercise self-control, leading employees to engage in unethical behavior (Zhang et al., 2019), counterproductive work behavior (Bazzy and Woehr, 2017; Wehrt et al., 2020), bullying behavior (McAllister and Perrewé, 2018), and destructive voice behavior (Mackey et al., 2020). Based on these findings, it is reasonable to believe that when good citizens are in a state of ego depletion, they may engage in UPB. This risk increases when organizational goals override ethical standards, creating moral ambiguity that enables UPB. This is because engaging in UPB helps good citizens rationalize their actions. It allows them to justify what they've done and avoid feelings of guilt or responsibility for the harm caused to others (Bandura, 2002). Specifically, when good citizens engage in unethical behavior for the benefit of the organization rather than for personal gain, they are more likely to downplay their own moral responsibility and experience less guilt and self-blame. At the same time, this framing enables them to morally justify their actions, leading them to believe that they neither deserve nor will receive blame or criticism. These cognitive distortions make good citizens more likely to engage in UPB when they are ego-depleted. Therefore, we propose Hypothesis 3.

*H3:* Ego depletion is positively associated with UPB.

Taken together, Hypotheses 1, 2, and 3 suggest that ego depletion may be a key mechanism through which OCB leads to UPB. When engaging in OCB depletes employees' self-control resources, they may become more likely to engage in UPB. Accordingly, Hypothesis 4 is advanced.

*H4:* Ego depletion mediates the relationship between OCB and UPB.

### 2.3 Moderating role of job insecurity

Job insecurity is a sense of threat that employees experience about the continuity and stability of their current work (Boswell et al., 2014; Shoss, 2017). It is not that employees have actually lost their jobs or face limited career development, but rather that they experience a sense of crisis due to uncertainty about their future employment (Jiang and Lavaysse, 2018). In response to this perceived threat, employees often intensify their work effort and display stronger commitment to signal their value to the organization and thereby secure their positions (Shoss, 2017). Accordingly, good citizens are required to make psychological and behavioral adjustments in order to maintain or improve their job performance under conditions of insecurity. In other words, job insecurity can significantly influence both the mindset and behavior of good citizens.

Job insecurity may aggravate the ego depletion associated with OCB among good citizens. Previous research suggests that job insecurity heightens employees' concern about their organizational standing and prompts them to invest more effort in maintaining or improving their performance at work (Huang et al., 2012; Lam et al., 2015). Under such conditions, OCB is less likely to be enacted as a purely voluntary and other-oriented behavior and more likely to be carried out under pressure related to self-protection and value maintenance. When employees engage in OCB in such a context, they are likely to invest greater self-regulatory effort to monitor their behavior, manage internal strain, and maintain a constructive and committed image. Moreover, under conditions of high job insecurity, good citizens may also engage in higher levels of OCB in an effort to demonstrate their value to the organization. Taken together, under conditions of high job insecurity, OCB may become both more resource-demanding and more frequent, thereby strengthening its positive relationship with ego depletion. Based on this reasoning, we propose the following hypothesis:

*H5:* Job insecurity positively moderates the effect of OCB on ego depletion.

**Figure 1:**
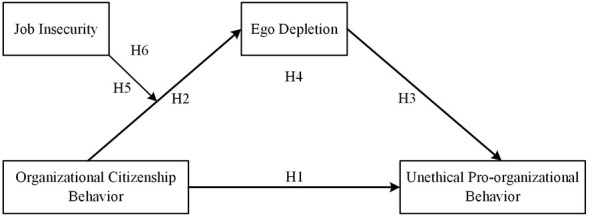
Hypothesized research model. H4 proposes that ego depletion mediates the relationship between OCB and UPB. H6 proposes that job insecurity moderates the indirect effect of OCB on UPB through ego depletion.

As previously discussed, engaging in OCB may drain good citizens' personal resources, which in turn may increase their likelihood of engaging in unethical conduct to continue supporting the organization. However, this mediating process is likely to depend on the level of job insecurity experienced by good citizens. Under high job insecurity, OCB is more likely to be carried out in a resource-demanding and self-protective manner, making ego depletion a more salient mechanism through which OCB leads to UPB. In contrast, when job insecurity is low, the mediating role of ego depletion is likely to be weaker. Therefore, job insecurity is expected to positively moderate the mediating role of ego depletion in the relationship between OCB and UPB. Based on this reasoning, we propose Hypothesis 6 and present the theoretical model of the study in Figure 1.

*H6:* Job insecurity positively moderates the mediating role of ego depletion in the relationship between OCB and UPB.

## 3 Materials and methods

### 3.1 Participants and procedures

This study conducted a questionnaire survey across 18 enterprises in China during July and August 2024. The participating organizations were mainly from industries more prone to UPB, including financial services, internet technology, real estate development, and marketing-oriented manufacturing. The majority of these firms were medium-sized or larger. Before the survey, we contacted the HR managers of the target companies to explain the research purpose and content. Upon obtaining their agreement, we scheduled the survey accordingly. Then, we requested them to randomly select participants from the employee list and gather them in a meeting room. During the meeting, we explained the purpose and process of the research to the potential participants, informing them that participation was voluntary and the survey would be anonymous. We assured them that the survey results would be kept strictly confidential and used only for academic research. In the end, we successfully recruited 450 employees to participate in the survey.

To reduce common method variance, a two-wave survey was conducted with a 20-day interval. Procedural remedies were also employed, such as randomizing the order of questionnaire items and using clear, concise, and neutral language to minimize response biases. The survey adopted a self-report format. Each participant was assigned a numerical code to ensure data matching across both waves. The first wave measured demographics, OCB, and job insecurity, while the second wave, conducted 20 days later, assessed ego depletion and UPB. After matching the responses, a total of 376 valid questionnaires were obtained, yielding an effective response rate of 83.56%. Among the 376 respondents, 41.5% were male and 59.5% were under the age of 35. Additionally, 60.4% held a bachelor's degree or above, and 48.9% had 10 or fewer years of work experience.

### 3.2 Measures

All measurement instruments in this study were adapted from previously validated scales. To ensure the reliability and validity of the original English scales in the Chinese context, we used a standard translation and back-translation procedure, made context-specific revisions, and consulted bilingual experts for review. All questionnaires adopted a 5-point Likert scale and were completed via self-report by employees. The specific scales used were as follows.

#### 3.2.1 Organizational citizenship behavior

OCB was measured using the 14-item scale developed by Williams and Anderson (1991). A sample item is “Attendance at work is above the norm,” rated from 1 (Never) to 5 (Very frequently). The scale demonstrated good internal consistency (Cronbach's α = 0.87).

#### 3.2.2 Ego depletion

The ego depletion scale developed by Deng et al. (2016) was used, consisting of 5 items. A sample item is: “I feel drained,” rated from 1 (Strongly disagree) to 5 (Strongly agree).The scale demonstrated a high level of internal consistency, with a Cronbach's α of 0.96.

#### 3.2.3 Job insecurity

We used the job insecurity scale developed by Wang et al. (2014), which consists of 5 items. A sample item is: “The thought of getting fired really scares you.” Responses were rated on a 5-point Likert scale ranging from 1 (Strongly disagree) to 5 (Strongly agree). The reliability coefficient was 0.83.

#### 3.2.4 Unethical pro-organizational behavior

We used the 6-item UPB scale developed by Umphress et al. (2010) to measure this construct. A sample item is “If it would help my organization, I would exaggerate the truth about my company's products or services to customers and clients”. Participants responded on a 5-point scale from “1 = Strongly disagree” to “5 = Strongly agree”. The scale had a Cronbach's alpha of 0.96.

#### 3.2.5 Control variables

Gender, age, work experience, and education level were included as demographic control variables. These variables were included to reduce potential confounding effects and to provide a cleaner test of the hypothesized relationships.

## 4 Results and discussion

### 4.1 Results

#### 4.1.1 Descriptive statistics

Table 1 presents the means, standard deviations, and correlation coefficients for the variables in this study. The results show that OCB is significantly positively correlated with ego depletion (*r* = 0.19), ego depletion is significantly positively correlated with UPB (*r* = 0.54), and OCB is also significantly positively correlated with UPB (*r* = 0.19). These results are consistent with the hypotheses of this study and provide a basis for further testing its theoretical model.

**Table 1:** Means, standard deviations, and correlations among study variables.

Variables	M	SD	1	2	3	4	5	6	7	8
1 Gender	1.59	0.49	1							
2 Age	2.64	0.96	0.05	1						
3 Education	2.75	1.04	0.04	−0.38^**^	1					
4 Years of work	3.45	1.39	−0.02	0.77^**^	−0.22^**^	1				
5 OCB	3.66	0.80	0.03	0.11^*^	−0.13^*^	0.11^*^	1			
6 ED	2.60	1.07	−0.17^**^	−0.21^**^	0.00	−0.16^**^	0.19^**^	1		
7 JI	2.58	1.03	−0.10	−0.12^*^	−0.10	−0.07	0.16^**^	0.64^**^	1	
8 UPB	2.68	1.08	−0.11^*^	−0.07	−0.13^*^	0.00	0.19^**^	0.54^**^	0.54^**^	1

*N* = 376; ^*^ *p* < 0.05 ^**^ *p* < 0.01 ^***^ *p* < 0.001; OCB, Organizational citizenship behavior; ED, Ego depletion; JI, Job insecurity; UPB, Unethical pro-organizational behavior.

#### 4.1.2 Measurement model

First, we used the Harman single-factor test to examine the common method bias in this study (Podsakoff and Organ, 1986). The results showed that the first factor accounted for 37.81% of the variance, which is below the 50% threshold, indicating that there was no serious common method bias. However, since the Harman single-factor test is not sensitive enough (Podsakoff et al., 2003), we also used Mplus7.0 to conduct a confirmatory factor analysis on four variables: OCB, ego depletion, unethical pro-organizational behavior, and job insecurity. The confirmatory factor analysis served two purposes: first, to further examine common method bias; second, to assess the discriminant validity of each variable. Table 2 shows that the confirmatory factor analysis revealed better fit indices for the four-factor model compared to the three-factor, two-factor, and single-factor models. This finding not only suggests that the four variables had good discriminant validity but also provides further evidence against serious common method bias in their measurement.

**Table 2:** Results of confirmatory factor analysis for the structural validity of variables.

Models	c^2^	*df*	c^2^*/df*	RMSEA	CFI	TLI
Four-factor model (OCB, ED, JI, UPB)	837.786	399	2.099	0.079	0.919	0.908
Three-factor model (OCB, ED+JI, UPB)	1159.774	402	2.885	0.093	0.887	0.874
Three-factor model (OCB+ED, JI, UPB)	1537.891	402	3.826	0.114	0.853	0.836
Two-factor model (OCB+ED, JI+UPB)	2375.020	404	5.879	0.202	0.716	0.686
One-factor model (OCB+ED+JI+UPB)	4620.753	405	11.409	0.263	0.418	0.360

“+” Indicates factors combined; OCB, Organizational citizenship behavior; ED, Ego depletion; JI, Job insecurity; UPB, Unethical pro-organizational behavior.

#### 4.1.3 Hypothesis tests

Hierarchical regression analyses were conducted to examine the proposed direct relationships among OCB, ego depletion, and UPB. UPB and ego depletion served as dependent variables in different models. Gender and the other control variables were entered first, followed by the focal predictors. As shown in Table 3, OCB had a significant positive effect on UPB (β = 0.18, *p* < 0.01, Model 4), supporting Hypothesis 1. OCB also exhibited a positive association with ego depletion (β = 0.21, *p* < 0.01, M2), providing support for Hypothesis 2. Further, ego depletion exerted a significant positive effect on UPB (β = 0.54, *p* < 0.01, M5), supporting Hypothesis 3.

**Table 3:** Regression results in this study.

Variable	ED			UPB		
	Model 1	Model 2	Model 3	Model 4	Model 5	Model 6
Gender	−0.16^*^	−0.16^*^	−0.09	−0.10	−0.01	−0.01
Age	−0.23^**^	−0.23^**^	−0.04	−0.26^**^	−0.14	−0.14^*^
Education	−0.08	−0.05	0.06	−0.17^**^	−0.15^*^	−0.14^*^
Years of work	0.00	−0.02	−0.09	0.14	0.16^*^	0.15^*^
OCB		0.21^**^	0.12^***^	0.18^**^		0.07
ED					0.54^**^	0.52^**^
JI			0.60^**^			
OCB ^*^ JI			0.12^**^			
***R** ^**2**^*	0.08	0.12	0.53	0.09	0.32	0.324
***F***	7.60^**^	9.99^**^	59.45^**^	6.99^**^	34.77^**^	29.54^**^
***DR** ^**2**^*	0.08	0.04	0.41	0.03	0.27	0.004

*N*=376; ^*^ *p* < 0.05 ^**^ *p* < 0.01 ^***^ *p* < 0.001; OCB, Organizational citizenship behavior; ED, Ego depletion; JI, Job insecurity; UPB, Unethical pro-organizational behavior.

Building on the support for Hypotheses 1, 2, and 3, we next examined the mediating role of ego depletion using the procedure proposed by Baron and Kenny (1986). Table 3 shows that, after controlling for ego depletion, the direct effect of OCB on UPB became non-significant (β = 0.07, *p* > 0.05, M6), suggesting a full mediating effect. This result was further corroborated by the bootstrapping method (Bolin, 2014). The indirect effect of OCB on UPB via ego depletion was 0.132, and the 95% confidence interval did not include zero [LLCI = 0.041, ULCI = 0.229], indicating that the mediating effect was statistically significant. Thus, Hypothesis 4 was supported.

**Figure 2:**
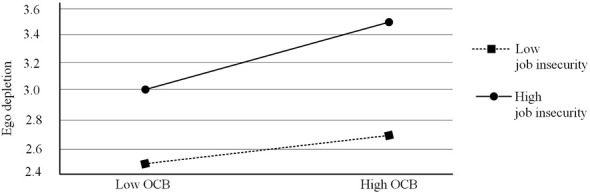
The moderating role of job insecurity in the relationship between OCB and ego depletion.

This study employed hierarchical regression to examine the moderating role of job insecurity in the relationship between OCB and ego depletion. Prior to the regression analysis, OCB, job insecurity, and were mean-centered to create the interaction terms. Table 3 shows that the interaction between OCB and job insecurity has a significant positive effect on ego depletion (β = 0.12, *p* < 0.01, M3), suggesting that job insecurity significantly strengthens the positive relationship between OCB and ego depletion. To further explore the moderating effect and clarify how OCB influences ego depletion under different levels of job insecurity, we examined their relationship under two conditions: high job insecurity (one standard deviation above the mean) and low job insecurity (one standard deviation below the mean). Figure 2 displays the results. The results indicate that when job insecurity is high, OCB significantly increases ego depletion (β = 0.21, *p* < 0.01). However, when job insecurity is low, the effect of OCB on ego depletion is non-significant (β = 0.02, *p* > 0.05). Therefore, hypothesis 5 is supported.

Based on Hypotheses 5, this study used the bootstrapping method (Bolin, 2014) to examine how job insecurity moderates the mediating effect of ego depletion in the relationship between OCB and UPB. As shown in Table 4, when job insecurity is high, the mediating effect of ego depletion is positive and significant (γ = 0.112, 95% CI [0.039, 0.186]). In contrast, when job insecurity is low, the mediating effect is not significant (γ = 0.012, 95% CI [−0.068, 0.079]). The difference between the two indirect effects is 0.100, which is statistically significant (95% CI [0.010, 0.204]). These findings support Hypothesis 6, indicating that employees with high job insecurity are more likely to experience ego depletion due to the personal resource demands of OCB, which in turn increases their likelihood of engaging in UPB. In contrast, employees with low job insecurity are less likely to follow this path.

**Table 4:** Mediated effect values of ego depletion at different levels of job insecurity.

Paths and effects	Effect	Standard error	Bootstrap's 95% CI	
OCB(X)→ED (M)→UPB (Y)			
High JI (+1SD)	0.112	0.037	0.039	0.186
Low JI (−1SD)	0.012	0.038	−0.068	0.079
Index of moderated mediation	0.100	0.049	0.010	0.204

OCB, Organizational citizenship behavior; ED, Ego depletion; JI, job insecurity; UPB, Unethical pro-organizational behavior.

### 4.2 Discussion

Drawing on the resource model of self-control, this study examined why good citizens may engage in UPB. The findings indicate that OCB, despite its generally positive image, may also lead employees to engage in unethical behavior for the benefit of the organization. This pattern differs from much of the prior research on the dark side of OCB, which has mainly emphasized self-interested negative outcomes or behaviors that ultimately harm the organization (e.g., Dalal et al., 2009; Bolino and Klotz, 2015; Yam et al., 2017). Our findings therefore suggest that the negative consequences of OCB may take a more ethically paradoxical form than has typically been recognized.

The findings also help clarify how this effect emerges. Although previous research has shown that OCB may entail hidden personal costs for employees (e.g., Bolino and Turnley, 2005; Bolino et al., 2015; Fu et al., 2022), less attention has been paid to how such costs may translate into unethical conduct. In the present study, engaging in OCB appears to consume employees' self-control resources, thereby increasing ego depletion and making good citizens more likely to engage in UPB as a way of continuing to support the organization. This suggests that the dark side of OCB may arise not only from instrumental or self-serving motives, but also from the depletion of personal resources during sustained extra-role effort.

In addition, this process is contingent on employees' level of job insecurity. When job insecurity is high, the indirect effect of OCB on UPB through ego depletion becomes more pronounced, whereas this effect is not observed when job insecurity is low. This finding indicates that the ethical consequences of OCB are not uniform, but depend on the broader work context in which extra-role behavior takes place. In this sense, the present study shows that whether OCB gives rise to UPB depends not only on resource depletion itself, but also on the degree of insecurity employees experience in their work environment.

## 5 Conclusion

This study demonstrates that OCB is not always beneficial. Although OCB is typically viewed as a constructive form of extra-role behavior, our findings show that it may also increase employees' likelihood of engaging in UPB. Specifically, OCB can drain employees' self-regulatory resources, heightening ego depletion and making them more prone to unethical acts intended to benefit the organization. In this sense, the negative side of OCB may be more ethically paradoxical than is often assumed. Moreover, this indirect effect is stronger under higher levels of job insecurity, suggesting that the tendency for OCB to translate into UPB through ego depletion is more likely to emerge when employees face greater insecurity about their jobs. These findings have important theoretical implications and offer meaningful insights for organizational practice and broader societal concerns.

## 6 Theoretical contributions

First, this study offers a novel perspective for deepening our understanding of the consequences of OCB. Employee behavior in the workplace is complex and multifaceted. It does not always manifest as bad behavior (e.g., Fox et al., 2012), nor is it consistently characterized by good behavior (e.g., Dalal et al., 2009; Yam et al., 2017). A more complete understanding of employee behavior therefore requires greater attention to its dynamic and potentially paradoxical nature. By examining the potential transition from OCB to UPB, this study provides a more nuanced framework for understanding employee behavioral patterns.

Second, this study identifies a previously underexplored dark side of OCB, thereby enriching the growing body of research on the unintended consequences of extra-role behavior. Unlike prior studies that have mainly emphasized self-interested harmful behaviors, our findings show that OCB may also give rise to unethical behavior undertaken to benefit the organization, despite its moral cost to employees. This finding contributes to the literature by identifying a distinct pathway to OCB's dark side, one driven by organizational concern rather than self-interest.

Finally, this study contributes by uncovering the psychological mechanism and boundary condition underlying this newly identified dark side of OCB. Specifically, ego depletion explains how OCB may lead to UPB, while job insecurity clarifies when this indirect effect is more likely to occur. By linking self-control theory to the connection between OCB and UPB, this study extends understanding of the process through which the negative consequences of OCB may emerge.

## 7 Practical and social implications

### 7.1 Managerial implications

First, it is important for managers to pay close attention to the sustainability of employee effort. Because OCB is not cost-free and consumes employees' self-control resources, organizations can adopt balanced work-rest arrangements, provide supportive recovery conditions, and foster a cooperative work climate to reduce ego depletion and prevent extra-role effort from becoming an ethical risk.

Second, organizations need to take active steps to reduce employees' job insecurity. Under conditions of job insecurity, good citizens may become more vulnerable to unethical pro-organizational behavior. Clearer career development plans, more transparent promotion criteria, and more stable expectations about employees' future prospects may help reduce this risk.

Third, strengthening employees' skills and professional confidence through targeted training is equally important. When employees feel more competent and in control of their work, they may be less likely to rely on unethical shortcuts to demonstrate their value and loyalty to the organization.

Fourth, effective communication and feedback channels also matter. Regular communication, one-to-one meetings, and confidential feedback mechanisms can reduce uncertainty, address employees' concerns in a timely manner, and strengthen their sense of support and security.

Finally, managers should ensure that expectations for employees to act as good soldiers remain within their capacity for self-control. Otherwise, such pressure may deplete employees' self-control resources and make unethical pro-organizational behavior more likely.

### 7.2 Social implications

First, the actions of good citizens may also carry broader social costs. When employees engage in unethical conduct for the benefit of the organization, the resulting harm often reaches beyond the workplace to customers, consumers, and the public. In such cases, short-term gains within the firm may come at the expense of social trust, fair exchange, and broader moral standards. The findings therefore suggest that the behavior of good citizens should be judged not only by the organizational value it creates, but also by the ethical costs it may generate beyond the workplace.

Second, the findings raise a broader ethical concern about the relationship between prosociality and morality. Behaviors that are typically praised as helpful, cooperative, or well-intentioned do not always produce socially desirable outcomes. Under conditions of strain and insecurity, employees may become more likely to justify ethically problematic actions as necessary for the organization. In this sense, the study points to the risk that prosociality, under pressure, may become disconnected from broader social responsibility.

Third, the present study highlights a good citizen paradox at the societal level. Employees who are usually seen as loyal, dedicated, and dependable may, under certain organizational conditions, become vulnerable to unethical conduct. This finding calls for a more cautious view of extreme loyalty and self-sacrificial effort, especially when they are embedded in demanding and insecure work environments.

## 8 Limitations and future research

First, this study faced the issue of same-source bias in variable measurement. Although this issue was mitigated to some extent through a two-wave survey design and procedural remedies such as anonymity and confidentiality, the potential for common method bias still exists. While statistical analysis indicated that the bias was not severe, future research should adopt multi-source and multi-time approaches, as recommended by Podsakoff et al. (2003), to further enhance the credibility of research findings.

Second, the narrow scope of the sample limits the external validity of the study's findings. In particular, the relatively small sample size may reduce both the statistical power and the generalizability of the findings. Moreover, because all data were collected within a single national or cultural context, the results may not be generalizable to other cultural or organizational environments. To further test the applicability of the findings, future research should increase both the diversity and size of the sample.

Finally, although this study examined how job insecurity moderates the mediating role of ego depletion between OCB and UPB, it did not further explore the organizational-level factors that might influence this mediation process. For example, organizational ethical pressure may deter ego-depleted employees from engaging in UPB. Future studies should investigate how organizational-level factors shape the behavior of good citizens who experience ego depletion as a result of OCB.

## Data Availability

The raw data supporting the conclusions of this article will be made available by the authors, without undue reservation.
